# Akt1 genetic variants confer increased susceptibility to thyroid cancer

**DOI:** 10.1530/EC-20-0311

**Published:** 2020-10-02

**Authors:** Thomas Crezee, Mirela Petrulea, Doina Piciu, Martin Jaeger, Jan W A Smit, Theo S Plantinga, Carmen E Georgescu, Romana Netea-Maier

**Affiliations:** 1 Department of Pathology, Radboud University Medical Center, Nijmegen, The Netherlands; 2 Radboud Institute for Molecular Life Sciences (RIMLS), Radboud University Medical Center, Nijmegen, The Netherlands; 3 Department of Endocrinology, Iuliu Hatieganu University of Medicine and Pharmacy, Cluj-Napoca, Romania; 4 Department of Nuclear Medicine and Endocrine Tumors, Institute of Oncology ‘Prof. Dr. Ion Chiricuta’, Cluj-Napoca, Romania; 5 Division of Endocrinology, Department of Internal Medicine, Radboud University Medical Center, Nijmegen, The Netherlands; 6 Endocrinology Clinic, Cluj County Emergency Hospital, Cluj-Napoca, Romania

**Keywords:** non-medullary thyroid cancer, susceptibility, PI3K/Akt/mTOR, genetic variation

## Abstract

The PI3K-Akt-mTOR pathway plays a central role in the development of non-medullary thyroid carcinoma (NMTC). Although somatic mutations have been identified in these genes in NMTC patients, the role of germline variants has not been investigated. Here, we selected frequently occurring genetic variants in *AKT1*, *AKT2*, *AKT3*, *PIK3CA* and *MTOR* and have assessed their effect on NMTC susceptibility, progression and clinical outcome in a Dutch discovery cohort (154 patients, 188 controls) and a Romanian validation cohort (159 patients, 260 controls). Significant associations with NMTC susceptibility were observed for *AKT1* polymorphisms rs3803304, rs2494732 and rs2498804 in the Dutch discovery cohort, of which the *AKT1* rs3803304 association was confirmed in the Romanian validation cohort. No associations were observed between PI3K-Akt-mTOR polymorphisms and clinical parameters including histology, TNM staging, treatment response and clinical outcome. Functionally, cells bearing the associated *AKT1* rs3803304 risk allele exhibit increased levels of phosphorylated Akt protein, potentially leading to elevated signaling activity of the oncogenic Akt pathway. All together, germline encoded polymorphisms in the PI3K-Akt-mTOR pathway could represent important risk factors in development of NMTC.

## Background

In recent years, the incidence of non-medullary thyroid cancer (NMTC) has steadily increased (1, 2, 3, 4). Although most NMTC patients have a favorable prognosis, 20–30% of patients with locally advanced or metastatic disease is confronted with long-term disease and increased risk of death as no curative treatment options are available (5, 6, 7).

The intracellular proteins PI3K, Akt and mTOR are part of a central signaling pathway in NMTC tumorigenesis by facilitating signal transduction to induce angiogenesis, metabolic reprogramming, proliferation and invasion of tumor cells (5, 8). Patients with Cowden’s disease, an autosomal dominant multiple hamartoma tumor syndrome caused by inactivating germline mutations in the *PTEN* gene and leading to constitutive activation of the PI3K-Akt-mTOR pathway, are at risk to develop several benign and malignant tumors, among which also NMTC (9, 10, 11). The important role of the PI3K-Akt-mTOR pathway in this respect has been confirmed by the identification of somatic driver mutations in the encoding *PI3KCA*, *AKT1*, *AKT2*, *AKT3* and *MTOR* genes in NMTC tumors, particularly in those having a poor prognosis (12, 13, 14, 15, 16). The approximate prevalence of these mutations varies from 1–2% in papillary thyroid cancer (PTC) up to 15–25% in anaplastic thyroid cancer (ATC) (6, 17). Furthermore, PI3K and mTOR targeted therapy has been observed to achieve beneficial effects by inhibiting NMTC proliferation and dedifferentiation, partly by activation of autophagy, providing the rationale for application of novel treatment modalities targeting this oncogenic pathway (18, 19, 20, 21, 22).

The PI3K kinase, encoded by the *PIK3CA* gene, is a protein directly downstream of receptor tyrosine kinases. Upon receptor activation, the signal is transmitted to PI3K and subsequently transferred to Akt by phosphorylation. Mammalian cells express three closely related Akt isoforms: Akt1 (PKBα), Akt2 (PKBβ) and Akt3 (PKBγ), all encoded by different genes. Whereas Akt1 is ubiquitously expressed, expression of Akt2 and Akt3 is restricted to certain tissues (23, 24). After phosphorylation of Akt isoforms by PI3K, the mTOR kinase is phosphorylated, leading to activation of downstream driving protein synthesis, proliferation and invasion of NMTC (5, 8, 25, 26).

Although somatic mutations have been identified at low frequencies, the role of germline variants in genes encoding PI3K, Akt and mTOR in the pathogenesis and clinical outcome of NMTC has not been studied so far. For the present study, we therefore hypothesized that PI3K, Akt and mTOR germline variants influence tumorigenesis and progression of NMTC in a similar fashion as somatically occurring mutations in the same genes.

## Materials and methods

### Study subjects

Patients with histologically confirmed NMTC who visited the Department of Endocrinology at the Iuliu Hatieganu University of Medicine and Pharmacy Cluj-Napoca or the Institute of Oncology Cluj-Napoca (IOCN), Romania and the outpatient clinic at the Division of Endocrinology of the Department of Internal Medicine, Radboud University Medical Center, Nijmegen, The Netherlands were asked to provide blood for genetic testing. In total, 154 consecutive Dutch NMTC patients (collected between 2009 and 2010, discovery cohort) and 159 Romanian NMTC patients (collected between 2014 and 2015, validation cohort) were enrolled in the study. Total thyroidectomy was performed in all cases in addition to modified radical lymph node neck dissections in patients with clinically or radiologically confirmed nodal metastases. NMTC diagnosis and histological classification was performed by experienced thyroid cancer pathologists. RAI (I-131) ablation of residual thyroid tissue was performed 4–6 weeks after surgery. Patients were repeatedly treated with RAI to reach remission, if indicated. Cured disease was defined according to institutional cut-off values of TSH stimulated thyroglobulin (Tg, <1 pmol/L in the Dutch patients and <0.04 ng/mL in the Romanian patients) in the absence of anti-Tg antibodies and no evidence of loco-regional disease or distant metastasis on the whole body iodine scans (WBS) and/or neck ultrasonographic examinations at 6–9 months after RAI ablation. Tumor recurrence was defined as new evidence of loco-regional disease or distant metastasis after successful primary therapy. Current disease status was defined as in remission in case of undetectable unstimulated Tg (according to the institutional cut-off) in the absence of anti-Tg antibodies and no evidence of loco-regional disease or distant metastases at the last follow-up visit. Persistent disease was defined as detectable Tg and/or evidence of loco-regional disease or distant metastases. Recurrent disease was defined as new evidence, biochemical (e.g. Tg becoming detectable after having been undetectable) and/or radiological, of loco-regional disease or distant metastases. Histological, clinical and follow-up data were retrieved from the patients’ medical records and are shown in Table 1. In addition, 188 Dutch and 260 Romanian healthy, genetically unrelated individuals, having no evidence of NMTC or other malignancies were recruited as population-based control subjects.

**Table 1:** Distribution of clinicopathological characteristics and treatment in the Dutch and Romanian non-medullary thyroid carcinoma (NMTC) cohorts.

Variables	Romanian NMTC cohort	Dutch NMTC cohort	*P*-values
	No. (%)		
Patients	159	154	
Age in years (mean ± s.d.)	52 (±14)	39 (±13)	0.24
Gender (F/M)	136/23	115/39	0.02
Tumor histology			
PTC	113 (71.1)	106 (68.8)	
FTC	37 (23.3)	37 (24.0)	0.75
FVPTC	9 (5.7)	10 (6.5)	
PDTC	0	1 (0.6)	
T-stage			
T1	77 (48.4)	45 (29.2)	
T2	26 (16.4)	51 (33.1)	0.001
T3	49 (30.8)	25 (16.2)	
T4	7 (4.4)	12 (7.8)	
Tx	0 (0)	21 (13.6)	
N-stage			
N0	95 (59.7)	80 (52.0)	
N1	40 (25.2)	51 (33.1)	0.28
Nx	24 (15.1)	23 (14.9)	
M-stage			
M0	122 (76.7)	106 (68.8)	
M1	11 (6.9)	4 (2.6)	0.01
Mx	26(16.4)	44 (28.6)	
Cumulative RAI activity (mCi)			
30–100	96 (60.4)	39 (25.3)	
100–200	28 (17.6)	55 (35.7)	0.001
≥ 200	35 (22.0)	60 (39.0)	
Persistent disease	65 (40.9)	67 (43.5)	0.64

FTC, follicular thyroid cancer; FVPTC, follicular-variant papillary thyroid cancer; PDTC, poorly differentiated thyroid cancer; PTC, papillary thyroid cancer; RAI, radioactive iodide.

### Genotyping

Single nucleotide polymorphisms (SNP) were selected based on population frequency, previously published associations with human diseases and/or known functional effects on protein function or gene expression (27, 28, 29, 30, 31, 32) (Table 2). After obtaining informed consent, blood was drawn from the cubital vein of participants into EDTA collection tubes and subjected to DNA extraction using the GeneJET™ Whole Blood Genomic DNA Purification Mini Kit (Fermentas, Thermo Fisher Scientific) according to the manufacturer’s instructions. Until further analysis, DNA samples were stored at −20°C. TaqMan SNP Genotyping assays (Life Technologies) designed with two specific probes and primers for each variant were utilized for genotyping the SNPs in *PIK3CA*, *AKT1*, *AKT2*, *AKT3* and *MTOR* (Table 2). Ten nanograms of genomic DNA were amplified by quantitative PCR (qPCR) in a 7300 Real-Time PCR System (Life Technologies), under standard conditions. The real-time PCR included an initial denaturation step at 95°C for 10 min, followed by 40 cycles at 95°C for 15 s and then at 60°C for 1 min. Quality control was performed by duplicating samples within and across plates and by the incorporation of positive and negative control samples.

**Table 2:** Selection of genotyped SNPs and TaqMan SNP genotyping assays for genotyping of polymorphisms in genes encoding components of the Akt-mTOR-PI3K pathway, including references of previous studies that revealed important genetic associations of these SNPs with cancer susceptibility or outcome.

Gene	SNP ID	Gene region	TaqMan SNP genotyping assay	References
*AKT1*	rs3803300	Promoter (5’ UTR)	C__27503538_10	Guo *et al.* (42), Lee *et al.* (43), Wang *et al.* (44), Kim *et al.* (45)
	rs3803304	Intron 12	C__27518787_10	Hildebrandt *et al.* (28), Pfisterer *et al.* (31), Pu *et al.* (32)
	rs2494732	Intron 12	C__16191608_10	Li *et al.* (29), Kim *et al.* 2012
	rs2498804	3’ UTR	C__11785058_10	Hildebrandt *et al.* (28), Li *et al.* (29), Pu *et al.* (32)
*AKT2*	rs3730050	Intron 2	C___7831393_10	Chen *et al.* (27)
*AKT3*	rs4132509	Intron 4	C__26719162_10	
*MTOR*	rs11121704	Intron 14	C__31720978_30	Shao *et al.* (46)
	rs2295080	Promoter (5’ UTR)	C__16189146_10	Shao *et al.* (46)
*PIK3CA*	rs2699887	Intron 1	C__16283198_10	Li *et al.* (29), Pu *et al.* (32)
	rs2677760	Promoter (5’ UTR)	C__16276690_10	Pande *et al.* (30)

### PBMC isolation and Western blotting

For isolation of peripheral blood mononuclear cells (PBMCs), venous blood was drawn from the cubital vein of healthy volunteers into 10 mL EDTA tubes (Monoject). The mononuclear cell fraction was obtained by density centrifugation of blood diluted 1:1 in pyrogen-free saline over Ficoll-Paque (Pharmacia Biotech). Cells were washed twice in saline and suspended in culture medium (RPMI, Invitrogen) supplemented with gentamicin 10 µg/mL, l-glutamine 10 mM and pyruvate 10 mM. Cells were counted in a Coulter counter (Coulter Electronics) and the number was adjusted to 5 x 10^6^ cells/mL. For Western blotting, cells were incubated with either culture medium (negative control) or with *E.coli* lipopolysaccharide (LPS, 100 ng/mL, Sigma) for 30 min, a well established activator of Akt signaling (33, 34). For western blotting of (phosphorylated) Akt protein, 5 x 10^6^ cells were lysed in 40 µL of lysis buffer (50 mM Tris (pH 7.4), 150 mM NaCl, 2 mM EDTA, 2 mM EGTA, 10% glycerol, 1% Triton X-100, 40 mM β-glycerophosphate, 50mM sodium fluoride, 200mM sodium vanadate, 10 mg/mL leupeptin, 10 mg/mL aprotinin, 1 mM pepstatin A, and 1 mM phenylmethylsulfonyl fluoride). The homogenate was frozen and then thawed and centrifuged at 4°C for 3 min at 14,000 ***g***, and the supernatant was mixed with a loading buffer containing dithiothreitol, incubated at 95°C for 15 min, and taken for western blot analysis. Equal amounts of protein were subjected to SDS-PAGE using 10% polyacrylamide gels. After SDS-PAGE, proteins were transferred to nitrocellulose membrane (0.2 mm). The membrane was blocked with 5% (wt/vol) milk powder in TBS/Tween 20 for 1 h at room temperature, followed by incubation overnight at 4°C with a pAkt S473 antibody (1:1000, Cell Signalling #9018) or total Akt antibody (1:1000, Cell Signalling #2938) in 5% BSA in TBS/Tween 20 or with a β-actin antibody (loading control, 1:1000, A2066; Sigma) in 5% milk powder in TBS/Tween 20. After overnight incubation, the blots were washed three times with TBS/Tween 20 and then incubated with horseradish peroxidase-conjugated swine anti-rabbit antibody at a dilution of 1:5000 in 5% (wt/vol) milk powder in TBS/Tween 20 for 1 h at room temperature. After being washed three times with TBS/Tween 20, the blots were developed with ECL (GE Healthcare) according to the manufacturer’s instructions.

### Statistical analysis

Genotypes and allele frequencies were calculated and the Hardy–Weinberg equilibrium was assessed using a goodness-of-fit *X^2^*-test for biallelic markers. The odds ratios (ORs) and 95% CI of the association between genotype frequencies and NMTC susceptibility in addition to clinicopathological characteristics and treatment outcomes were analyzed using logistic regression models. In addition, χ^2^ analysis and Fisher’s exact test were applied to determine whether tumor size, cumulative RAI activity (subdivided as 30–100 mCi (1.1–3.8 GBq), 101 –200 mCi (3.8–7.4 GBq) or ≥200 mCi (>7.4 GBq)) and disease status after thyroidectomy plus radio-ablation were associated with the genotype of the analyzed genes. All statistical analyses were carried out with SPSS for statistical computing and graphics. Differences in protein amounts detected by western blot were analyzed using the Mann–Whitney *U* test. Overall, statistical tests were two-sided and a *P*-value below 0.05 was considered statistically significant.

## Results

### PI3K-Akt-mTOR pathway SNPs and susceptibility to NMTC

To assess the effects of genetic variation in PI3K-Akt-mTOR genes on susceptibility to NMTC, several SNPs were selected based on previously published associations with human diseases and/or known functional effects on protein function or gene expression. The genotypes corresponding to these SNPs were determined in the Dutch discovery cohort (154 patients, 188 healthy controls) and in the Romanian validation cohort (159 patients, 260 healthy controls). Table 1 summarizes the main clinical and demographical characteristics of the selected Dutch and Romanian NMTC patients. Distribution of gender, tumor size staging, metastasis staging and cumulative RAI activity were significantly different between the Dutch and Romanian patient cohorts. The distribution of *PIK3CA*, *AKT1*, *AKT2*, *AKT3* and *MTOR* genotypes among the Dutch and Romanian cohorts are presented in Tables 3 and 4, respectively. These results demonstrate the association of the rs3803304, rs2494732 and rs2498804 polymorphisms in *AKT1* with NMTC susceptibility in the Dutch discovery cohort by applying different genetic association models. Importantly, in the Romanian validation cohort the *AKT1* rs3803304 polymorphism was confirmed as genetic risk factor for NMTC in the dominant model. Of note, genotype frequencies in both NMTC patients and controls study populations were in accordance with that expected under the Hardy–Weinberg equilibrium.

**Table 3:** Genetic distribution of genetic variants in PI3K, Akt and mTOR genes in the Dutch cohort of thyroid carcinoma patients (*n* = 154) and healthy controls (*n* = 188).

Gene	Polymorphism	Allelic distribution (reference genotype)				*P*-values^a^ and OR^b^ (95% CI)		
						Dose-dependent	Dominant	Recessive
*AKT1*	rs3803300		GG^c^	GA	AA	0.123	0.749	0.055
		Patients	125 (81.2%)	26 (16.9%)	3 (1.9%)		1.092 (0.637–1.871)	N/A
		Controls	150 (79.8%)	38 (20.2%)	0 (0%)			
*AKT1*	rs3803304		GG^c^	GC	CC	0.034	0.035	0.040
		Patients	75 (48.7%)	65 (42.2%)	14 (9.1%)		1.587 (1.032–2.439)	1.608 (1.008–2.564)
		Controls	113 (60.1%)	68 (36.2%)	7 (3.7%)			
*AKT1*	rs2494732		TT^c^	TC	CC	0.057	0.031	0.081
		Patients	42 (27.3%)	74 (48.1%)	38 (24.7%)		1.656 (1.044–2.625)	1.264 (0.971–1.645)
		Controls	72 (38.3%)	84 (44.7%)	32 (17.0%)			
*AKT1*	rs2498804		GG^c^	GT	TT	0.049	0.119	0.020
		Patients	64 (41.6%)	63 (40.9%)	27 (17.5%)		1.406 (0.915–2.160)	1.462 (1.057–2.024)
		Controls	94 (50.0%)	77 (41.0%)	17 (9.0%)			
*AKT2*	rs3730050		GG^c^	GA	AA	0.961	0.939	0.780
		Patients	78 (50.6%)	59 (38.3%)	17 (11.0%)		1.016 (0.664–1.558)	1.050 (0.743–1.486)
		Controls	96 (51.1%)	73 (38.8%)	19 (10.1%)			
*AKT3*	rs4132509		CC^c^	CA	AA	0.760	0.474	0.703
		Patients	95 (61.7%)	52 (33.8%)	7 (4.5%)		1.175 (0.755–1.828)	1.110 (0.650–1.894)
		Controls	123 (65.4%)	58 (30.9%)	7 (3.7%)			
*MTOR*	rs11121704		TT^c^	TC	CC	0.996	0.931	0.998
		Patients	90 (58.4%)	55 (35.7%)	9 (5.8%)		1.019 (0.662–1.570)	1.001 (0.636–1.575)
		Controls	109 (58.0%)	68 (36.2%)	11 (5.9%)			
*MTOR*	rs2295080		TT^c^	TG	GG	0.936	0.991	0.731
		Patients	81 (52.6%)	63 (40.9%)	10 (6.5%)		1.002 (0.654–1.536)	1.076 (0.707–1.639)
		Controls	99 (52.7%)	75 (39.9%)	14 (7.4%)			
*PIK3CA*	rs2699887		GG^c^	GA	AA	0.482	0.975	0.249
		Patients	83 (53.9%)	59 (38.3%)	12 (7.8%)		1.007 (0.657–1.544)	1.297 (0.830–2.024)
		Controls	101 (53.7%)	78 (41.5%)	9 (4.8%)			
*PIK3CA*	rs2677760		TT^c^	TC	CC	0.870	0.597	0.901
		Patients	30 (19.5%)	97 (63.0%)	27 (17.5%)		1.153 (0.680–1.953)	1.018 (0.768–1.350)
		Controls	41 (21.8%)	115 (61.2%)	32 (17.0%)			

^a^Generated by Chi-square analysis; ^b^Calculated by binary logistic regression; ^c^Reference genotype.

N/A, not applicable.

**Table 4:** Genetic distribution of genetic variants in PI3K, Akt and mTOR genes in the Romanian cohort of thyroid carcinoma patients (*n* = 159) and healthy controls (*n* = 260).

Gene	Polymorphism	Allelic distribution (reference genotype)				*P*-values^a^ and OR^b^ (95% CI)		
						Dose-dependent	Dominant	Recessive
*AKT1*	rs3803300		GG^c^	GA	AA	0.052	0.251	0.066
		Patients	123 (77.4%)	31 (19.5%)	5 (3.1%)		1.309 (0.826–2.073)	2.045 (0.896–4.673)
		Controls	188 (72.3%)	70 (26.9%)	2 (0.8%)			
*AKT1*	rs3803304		GG^c^	GC	CC	0.072	0.022	0.580
		Patients	76 (47.8%)	67 (42.1%)	16 (10.1%)		1.587 (1.066–2.358)	1.100 (0.784–1.543)
		Controls	154 (59.2%)	84 (32.3%)	22 (8.5%)			
*AKT1*	rs2494732		TT^c^	TC	CC	0.719	0.893	0.478
		Patients	45 (28.3%)	75 (47.2%)	39 (24.5%)		1.031 (0.664–1.599)	1.088 (0.861–1.374)
		Controls	72 (27.7%)	132 (50.8%)	56 (21.5%)			
*AKT1*	rs2498804		GG^c^	GT	TT	0.116	0.073	0.104
		Patients	61 (38.4%)	70 (44.0%)	28 (17.6%)		1.443 (0.965–2.155)	1.256 (0.952–1.658)
		Controls	123 (47.3%)	106 (40.8%)	31 (11.9%)			
*AKT2*	rs3730050		GG^c^	GA	AA	0.469	0.226	0.883
		Patients	80 (50.3%)	65 (40.9%)	14 (8.8%)		1.277 (0.860–1.897)	1.026 (0.727–1.450)
		Controls	115 (44.2%)	121 (46.5%)	24 (9.2%)			
*AKT3*	rs4132509		CC^c^	CA	AA	0.315	0.996	0.140
		Patients	104 (65.4%)	48 (30.2%)	7 (4.4%)		1.001 (0.661–1.516)	1.531 (0.856–2.747)
		Controls	170 (65.4%)	85 (32.7%)	5 (1.9%)			
*MTOR*	rs11121704		TT^c^	TC	CC	0.143	0.049	0.520
		Patients	81 (51.0%)	67 (42.1%)	11 (6.9%)		1.493 (1.002–2.222)	1.143 (0.760–1.718)
		Controls	158 (60.8%)	88 (33.8%)	14 (5.4%)			
*MTOR*	rs2295080		TT^c^	TG	GG	0.150	0.331	0.055
		Patients	84 (52.8%)	56 (35.2%)	19 (11.9%)		1.218 (0.819–1.812)	1.393 (0.988–1.965)
		Controls	150 (57.7%)	93 (35.8%)	17 (6.5%)			
*PIK3CA*	rs2699887		GG^c^	GA	AA	0.622	0.937	0.364
		Patients	96 (60.4%)	57 (35.8%)	6 (3.8%)		1.016 (0.679–1.522)	1.249 (0.770–2.027)
		Controls	158 (60.8%)	87 (33.5%)	15 (5.8%)			
*PIK3CA*	rs2677760		TT^c^	TC	CC	0.360	0.177	0.448
		Patients	29 (18.2%)	103 (64.8%)	27 (17.0%)		1.404 (0.857–2.299)	1.110 (0.847–1.456)
		Controls	62 (23.9%)	161 (61.9%)	37 (14.2%)			

^a^Generated by Chi-square analysis; ^b^Calculated by binary logistic regression; ^c^Reference genotype.

N/A, not applicable.

### PI3K-Akt-mTOR pathway SNPs and clinical outcome of NMTC

Within the NMTC study populations recruited in The Netherlands and Romania, the impact of *PIK3CA*, *AKT1*, *AKT2*, *AKT3* and *MTOR* genotypes on the clinical postoperative treatment response and outcome of NMTC patients was investigated. These analyses revealed that none of the investigated polymorphisms in the PI3K-Akt-mTOR pathway were associated with worse clinical manifestation of NMTC in any of the cohorts regarding histology, TNM staging, RAI treatment response and clinical outcome (Supplementary Tables 1 and 2, see section on supplementary materials given at the end of this article).

### Functional consequences of ***AKT1*** rs3803304 polymorphism for pAkt and total Akt protein expression in PBMCs

**Figure 1:**
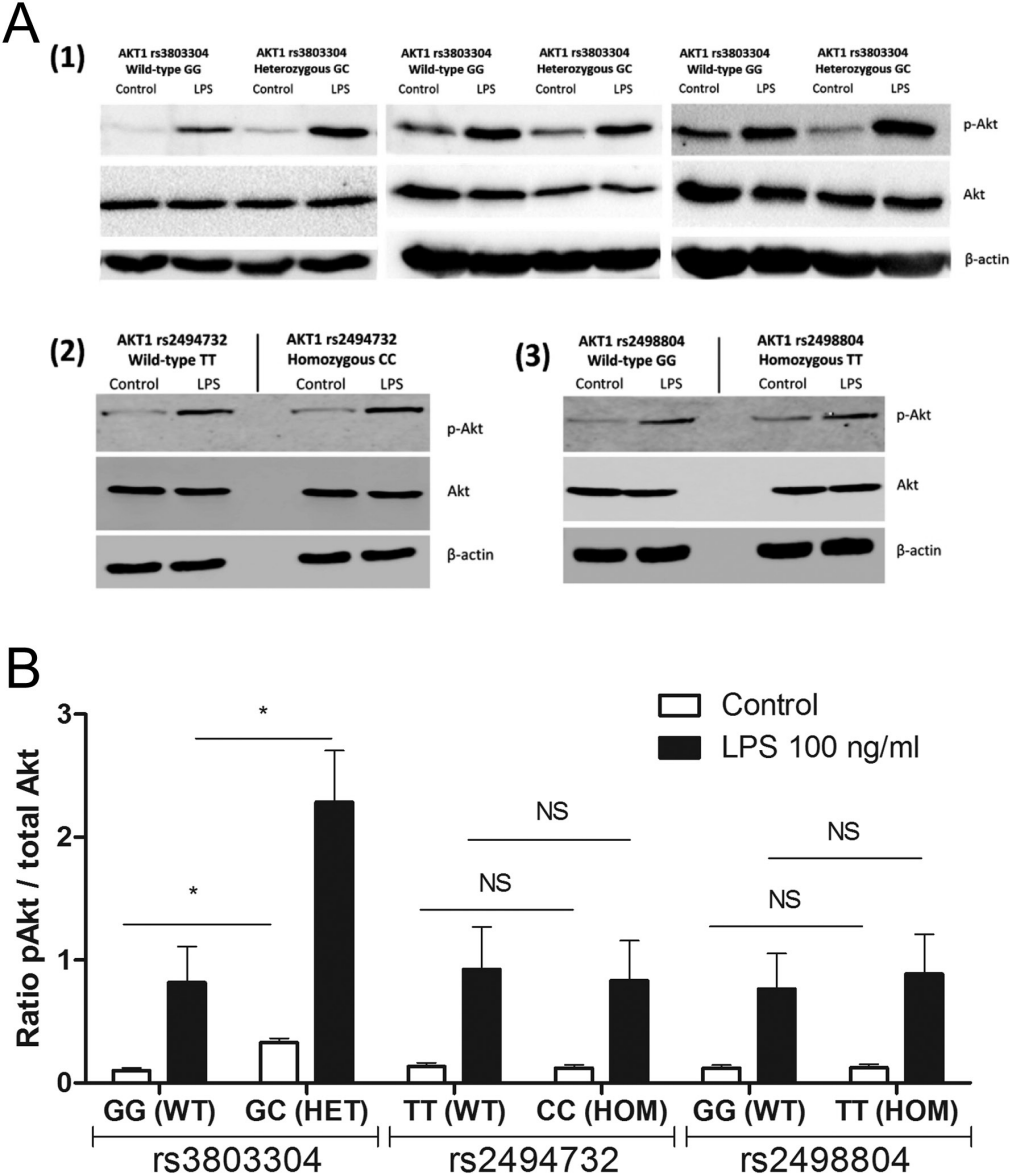
(A) Western blot detection of Akt and p-Akt proteins in PBMCs from individuals either (1) WT or heterozygous for *AKT1* rs3803304 polymorphism (*n* = 3), (2) WT or homozygous for *AKT1* rs2494732 polymorphism (*n* = 1) or (3) WT or homozygous for *AKT1* rs2498804 polymorphism (*n* = 1). Cells were left untreated or stimulated with 100 ng/mL LPS for 30 min. Detection of β-actin served as loading control. Representative of four independent experiments and per experiment two donors per genotype group. Figures represent cropped images. (B) Quantification of pAkt/Akt ratios obtained by Western blots as depicted in (A). Data are mean ± s.e.m. (*P-values <0.05) are generated by Mann–Whitney *U* tests, *n* = 4).

The observed genetic associations of the *AKT1* rs3803304 polymorphism with NMTC susceptibility in both cohorts and of the *AKT1* rs2494732 and rs2498804 polymorphisms in only the Dutch cohort suggest that these polymorphisms could influence Akt expression or function. To assess the potential functional effects of these polymorphisms, healthy individuals were stratified for *AKT1* rs3803304, *AKT1* rs2494732 or *AKT1* rs2498804 genotypes and their PBMCs were tested for differential levels of phosphorylated and total Akt protein, either in the unstimulated condition or after treatment with LPS for 30 min. Since individuals homozygous for the *AKT1* rs3803304 minor allele are rare, only subjects either WT or heterozygous for the *AKT1* rs3803304 minor allele could be included. The results indicate that no significant differences were apparent in total Akt expression between the genotypes. Interestingly, however, the amount of phosphorylated Akt is elevated in the individuals heterozygous for the *AKT1* rs3803304 risk allele in both the unstimulated and LPS-stimulated condition as compared to WT subjects. In contrast, no differences in phosphorylated Akt are apparent between WT and homozygous *AKT1* rs2494732 or *AKT1* rs2498804 genotypes in either unstimulated or LPS-stimulated conditions (Fig. 1A and B).

## Discussion

In recent years, the major oncogenic pathways have been elucidated that drive tumor initiation and progression in NMTC, which mainly comprise the RAS-RAF-MEK-ERK and PI3K-AKT-mTOR signaling pathways (6, 35). Although the contribution of somatic mutations in the activation of oncogenic signaling through these pathways has been well-established, the influence of germline variants on these pathways is still poorly characterized. The present study was performed to assess the effect of germline variants in the oncogenes *PIK3CA*, *AKT1*, *AKT2*, *AKT3* and *MTOR* on NMTC susceptibility and clinical outcome. For this, a Dutch discovery cohort and a Romanian validation cohort were gathered, consisting of NMTC patients and healthy unrelated controls, that allowed the assessment of potential genetic associations of selected germline polymorphisms with susceptibility to NMTC and with its clinical presentation, treatment response and patient outcome.

Interestingly, by the present study polymorphisms in the *AKT1* gene were shown to be significantly associated with NMTC susceptibility in the Dutch discovery cohort and the Romanian validation cohort. Whereas three *AKT1* polymorphisms (rs3803304, rs2494732 and rs2498804) were identified as statistically significant in the Dutch cohort, one of these, the rs3803304 polymorphism, was confirmed in the Romanian cohort. As opposed to polymorphisms in genes encoding PI3K, Akt2, Akt3 and mTOR, these results suggest major consequences of *AKT1* polymorphisms for Akt function, especially rs3803304, in modulating the activity of the PI3K-Akt-mTOR signaling pathway. Importantly, this genetic association was observed in both cohorts despite the statistically significant differences in clinical parameters between the Dutch and Romanian patient cohorts listed in Table 1. For the other *AKT1* polymorphisms that were only significantly associated with NMTC susceptibility in the Dutch cohort, it cannot be excluded that the lack of association with NMTC susceptibility in the Romanian cohort could be clarified by the differential distribution of these clinical parameters.

Additional analyses were performed to assess whether the selected polymorphisms are associated with clinical parameters including histology, TNM staging, cumulative RAI activity and remission rates. No statistically significant differences were observed, suggesting that *AKT1* polymorphisms are involved in tumor initiation rather than in processes of tumor progression, RAI therapy resistance and disease persistence.

Of note, with solid statistical significance the Romanian cohort received twice more low and medium RAI activities as compared to the Dutch patients, however is not associated with differences in remission rates. Again, this suggests that *AKT1* polymorphisms are involved in tumor initiation rather than in processes of tumor progression, despite the I-131 activities used for treatment. Also, there are differences in radiation exposure; Romania was among the countries affected by Chernobyl fallout and considering the average age of the Romanian patients (52 ± s.d.) this would be interesting to be studied in relation to the *AKT1* polymorphisms.

By functional assays it was demonstrated that the *AKT1* rs3803304 polymorphism, in contrast to the *AKT1* rs2494732 and *AKT1* rs2498804 polymorphisms, has a major effect on Akt phosphorylation, both in the naïve state and upon activation of the PI3K-Akt-mTOR pathway; cells bearing the heterozygous GC genotype, with the C allele conferring increased NMTC risk, exhibited elevated levels of phosphorylated Akt as compared to the GG genotype. These major functional consequences of the *AKT1* rs3803304 polymorphism provides mechanistic insights into the observed genetic association. Furthermore, these findings support the current evidence that the PI3K-Akt-mTOR signaling pathway plays a major role in NMTC and could represent a promising strategy for targeted treatment (5, 19, 25, 36).

Previously, the *AKT1* rs3803304 polymorphism has been demonstrated to also influence susceptibility, disease progression or clinical outcome of head and neck squamous cell carcinoma, lung carcinoma and esophageal carcinoma, indicating its major functional and clinical implications. In case susceptibility analyses were performed in these studies, the *AKT1* rs3803304 minor allele was associated with increased cancer susceptibility in all, confirming the contribution of the minor C allele in the etiology of multiple cancer types (28, 31, 32, 37, 38). Additionally to these reports, the present study provides mechanistic insights into the genetic association by linking the *AKT1* rs3803304 minor C allele with elevated Akt phosphorylation. The exact biological consequences of the polymorphism and whether it promotes Akt phosphorylation or inhibits Akt dephosphorylation remains to be determined.

Although the heterozygous GC genotype, and most likely also the homozygous CC genotype, are demonstrated to predispose to development of NMTC by inducing hyperactivation of the PI3K-Akt-mTOR pathway upon the encounter of activating stimuli, it should be emphasized that this germline genetic variant is not capable of evoking thyroid tumorigenesis by itself because of limited genetic penetrance, but rather represents a risk modifier. Within the context of NMTC, these activating stimuli could range from growth factors to metabolites (e.g. lactate) and inflammatory molecules (e.g. danger-associated molecular patterns and pro-inflammatory cytokines) produced by the tumor microenvironment, triggering either receptor tyrosine kinases, metabolic receptors, toll-like receptors or cytokine receptors expressed by follicular thyroid (tumor) cells (39, 40, 41).

In conclusion, the present study suggests that germline variants in the *AKT1* gene are an important risk factor in the etiology of NMTC, reinforcing the clinical utility of kinase inhibitors targeting the PI3K-Akt-mTOR pathway to abrogate pathological signaling driving the NMTC malignant process.

## Supplementary Material

Supplementary Table 1. Association of PI3K-Akt-mTOR polymorphisms with clinical parameters with OR (based on dominant model) and P-values (χ2, gene dose-dependent model): Dutch cohort.

Supplementary Table 2. Association of PI3K-Akt-mTOR polymorphisms with clinical parameters with OR (based on dominant model) and P-values (χ2, gene dose-dependent model): Romanian Cohort.

## Declaration of interest

The authors declare that there is no conflict of interest that could be perceived as prejudicing the impartiality of the research reported.

## Funding

This work was supported by the European Social Fundhttp://dx.doi.org/10.13039/501100004895, Human Resources Development Operational Programme 2007–2013, project no. POSDRU/159/1.5/S/138776. T S P was supported by a Veni grant of the Netherlands Organization for Scientific Research (NWO; 016.136.065) and by the Alpe d’HuZes fund of the Dutch Cancer Society (KUN2014-6728).

## Ethics approval and consent to participate

The study has been performed in accordance with the Declaration of Helsinki and approval was obtained from the Ethics Committees of Iuliu Hatieganu University of Medicine and Pharmacy Cluj-Napoca, Romania and Radboud University Medical Centre, Nijmegen, The Netherlands. Informed consent has been obtained from each patient or subject after full explanation of the purpose and nature of all procedures used.

## Consent for publication

All authors are aware of and agree to the submission and all authors have contributed to the work described sufficiently to be named as authors. Any other person or body with an interest in the manuscript is aware of the submission and agrees to it.

## Availability of data and materials

All raw data and study materials are available upon request.

## Author contribution statement

T S P, M S P, T C and M J performed the experiments and data analysis. T S P, M S P, J W S, R N M, D P, C E G, T C and M J designed the study and wrote the manuscript. All authors read and approved the final manuscript. All authors had full access to all of the data in the study and take responsibility for the integrity of the data and the accuracy of the data analysis.
