# Supplementary material for: Akt1 genetic variants confer increased susceptibility to thyroid cancer
Source: Endocr Connect. 2020 Oct 2;9(11):1065–74. doi: 10.1530/EC-20-0311 (PMC7774771; doi:10.1530/EC-20-0311)
Supplement: Supplementary Table 1. Association of PI3K-Akt-mTOR polymorphisms with clinical parameters with OR (based on dominant model) and P-values (χ2, gene dose-dependent model): Dutch cohort. [file supplementary_table_1.pdf]

Supplementary Table 1. Association of PI3K-Akt-mTOR polymorphisms with clinical parameters with OR (based on dominant model) and P-values (χ^2, gene dose-dependent model): Dutch cohort.

Variable		Total (±SD or %)	Unit	AKT1 rs3803300	AKT1 rs3803304	AKT1 rs2494732	AKT1 rs2498804	AKT2 rs3730050	AKT3 rs4132509	MTOR rs11121704	MTOR rs2295080	PIK3CA rs2699887	PIK3CA rs2677760
Patients (number)		154											
Age at diagnosis, mean years (± SD)		39 (± 13)											
Gender (Female/Male)		115/39	P	0.67	0.42	0.59	0.61	0.79	0.59	0.64	0.66	0.54	0.52
Tumor histology	PTC	106 (68.8%)	P	0.86	0.84	0.63	0.84	0.11	0.14	0.51	0.55	0.38	0.61
	FTC	37 (24.0%)											
	FVPTC	10 (6.5%)											
	PDTC	1 (0.6%)											
Treatment response	RAI sessions 0-1	91 (59.0%)	OR	1.24	1.23	1.45	1.16	1.23	1.42	1.23	1.27	1.25	1.64
	RAI sessions ≥ 2	63 (41.0%)	P	0.64	0.48	0.12	0.18	0.40	0.11	0.20	0.15	0.35	0.47
	Cum. RAI ≤ 3.7 GBq	39 (25.3%)	OR	1.25	1.07	1.35	1.10	1.23	1.49	1.37	1.48	1.02	1.31
	Cum. RAI 3.8-7.4 GBq	55 (35.7%)	P	0.30	0.49	0.29	0.57	0.87	0.29	0.32	0.22	0.36	0.39
	Cum. RAI > 7.4 GBq	60 (39.0%)											
	Remission after RAI ablation	87 (56.5%)	OR	1.09	1.52	1.60	1.30	1.14	1.86	1.02	1.01	1.00	1.26
	Persistence after RAI ablation	67 (43.5%)	P	0.95	0.47	0.44	0.48	0.92	0.17	0.28	0.29	0.32	0.58
Tumor stageing	T1	45 (29.2%)	P	0.58	0.71	0.59	0.48	0.49	0.70	0.98	0.68	0.88	0.75
	T2	51 (33.1%)											
	T3	25 (16.2%)											
	T4	12 (7.8%)											
	Tx	21 (13.6%)											
Node stageing	N0	80 (52.0%)	OR	1.32	1.58	1.01	1.22	1.05	1.12	1.16	1.04	1.01	1.18
	N1	51 (33.1%)	P	0.55	0.10	0.16	0.12	0.93	0.98	0.20	0.20	0.71	0.62
	Nx	23 (14.9%)											
Metastasis stageing	M0	106 (68.8%)	OR	1.07	1.12	1.06	1.03	1.02	1.01	1.11	1.02	1.26	1.16
	M1	4 (2.6%)	P	0.88	0.36	0.97	0.90	0.48	0.91	0.59	0.62	0.14	0.72
	Mx	44 (28.6%)											

PTC: papillary thyroid carcinoma; FTC: follicular thyroid carcinoma; FVPTC: follicular variant papillary thyroid carcinoma; PDTC: poorly differentiated thyroid carcinoma. RAI: radioactive iodide. Cum. RAI: cumulative radioactive iodide dose
