# Supplementary material for: Akt1 genetic variants confer increased susceptibility to thyroid cancer
Source: Endocr Connect. 2020 Oct 2;9(11):1065–74. doi: 10.1530/EC-20-0311 (PMC7774771; doi:10.1530/EC-20-0311)
Supplement: Supplementary Table 2. Association of PI3K-Akt-mTOR polymorphisms with clinical parameters with OR (based on dominant model) and P-values (χ2, gene dose-dependent model): Romanian Cohort. [file supplementary_table_2.pdf]

Supplementary Table 2. Association of PI3K-Akt-mTOR polymorphisms with clinical parameters with OR (based on dominant model) and P-values (χ^2, gene dose-dependent model): Romanian Cohort.

Variable		Total (±SD or %)	Unit	AKT1 rs3803300	AKT1 rs3803304	AKT1 rs2494732	AKT1 rs2498804	AKT2 rs3730050	AKT3 rs4132509	MTOR rs11121704	MTOR rs2295080	PIK3CA rs2699887	PIK3CA rs2677760
Patients (number)		159											
Age at diagnosis, mean years (± SD)		52 (±14)											
Gender (Female/Male)		136/23	P	0.20	0.70	0.75	0.67	0.15	0.65	0.40	0.41	0.64	0.72
	PTC	113 (71.1%)	P	0.37	0.63	0.54	0.79	0.33	0.26	0.94	0.57	0.74	0.64
	FTC	37 (23.3%)											
	FVPTC	9 (5.7%)											
Treatment response	RAI sessions 0-1	118 (74.2%)	OR	1.67	1.80	1.49	1.42	1.28	1.05	1.32	1.35	1.17	1.27
	RAI sessions ≥ 2	41 (25.8%)	P	0.61	0.03	0.32	0.04	0.58	0.93	0.52	0.50	0.51	0.64
	Cum. RAI ≤ 3.7 GBq	96 (60.4%)	OR	1.71	1.54	1.30	1.11	1.04	1.30	1.83	1.75	1.18	1.11
	Cum. RAI 3.8-7.4 GBq	28 (17.6%)	P	0.19	0.23	0.34	0.79	0.91	0.19	0.11	0.14	0.68	0.87
	Cum. RAI > 7.4 GBq	35 (22.0%)											
	Remission after RAI ablation	119 (74.8%)	OR	1.01	1.03	1.07	1.16	1.29	1.37	1.15	1.05	1.02	1.16
	Persistence after RAI ablation	40 (25.2%)	P	0.96	0.86	0.38	0.80	0.68	0.12	0.93	0.87	0.87	0.91
Tumor stageing	T1	77 (48.4%)	P	0.33	0.19	0.54	0.30	0.03	0.44	0.83	0.88	0.25	0.58
	T2	26 (16.4%)											
	T3	49 (30.8%)											
	T4	7 (4.4%)											
	Tx	0 (0%)											
Node stageing	N0	95 (59.7%)	OR	1.42	1.66	1.46	1.60	2.11	1.61	1.09	1.06	1.42	1.74
	N1	40 (25.2%)	P	0.88	0.45	0.12	0.47	0.34	0.79	0.36	0.18	0.04	0.31
	Nx	24 (15.1%)											
Metastasis stageing	M0	122 (76.7%)	OR	1.25	1.32	1.06	1.20	2.59	1.65	1.75	1.95	1.67	1.44
	M1	11 (6.9%)	P	0.91	0.85	1.00	0.63	0.15	0.91	0.05	0.13	0.35	0.61
	Mx	26 (16.4%)											

PTC: papillary thyroid carcinoma; FTC: follicular thyroid carcinoma; FVPTC: follicular variant papillary thyroid carcinoma; RAI: radioactive iodide; Cum. RAI: cumulative radioactive iodide dose.
